# Dietary patterns and metabolic syndrome in a Japanese working population

**DOI:** 10.1186/1743-7075-10-30

**Published:** 2013-03-27

**Authors:** Shamima Akter, Akiko Nanri, Ngoc Minh Pham, Kayo Kurotani, Tetsuya Mizoue

**Affiliations:** 1 Department of Epidemiology and Prevention, Clinical Research Center, National Center for Global Health and Medicine, Tokyo, Japan

**Keywords:** Cross-sectional study, Metabolic syndrome, Dietary pattern, Japanese

## Abstract

**Background:**

Metabolic syndrome has become a major public health concern, but the role of diet in the etiology of this syndrome is not well understood. This study investigated the association between major dietary patterns and prevalence of metabolic syndrome in a Japanese working population.

**Methods:**

This cross-sectional study was conducted among 460 municipal employees (284 men and 176 women), aged 21–67 years, who participated in a health survey at the time of periodic checkup. Dietary patterns were derived by using the principal component analysis of the consumption of 52 food and beverage items, which were assessed by a validated brief diet history questionnaire. Metabolic syndrome was defined according to the modified NCEP-ATP III criteria. Logistic regression was used to examine the association between dietary patterns and metabolic syndrome with adjustment of potential confounding variables.

**Results:**

Three dietary patterns were identified. Westernized breakfast pattern characterized by high intakes of bread, confectionaries, and milk and yogurt but low intakes of rice and alcoholic beverages was inversely associated with prevalence of metabolic syndrome and high blood pressure (*P* for trend = 0.02 and 0.049, respectively). Animal food pattern characterized by high intakes of fish and shellfish, meat, processed meat, mayonnaise, and egg was not associated with prevalence of metabolic syndrome, but was positively associated with high blood glucose (*P* for trend = 0.03). Healthy Japanese dietary pattern characterized by vegetables and fruits, soy products, mushrooms, and green tea was not appreciably associated with prevalence of metabolic syndrome or its components.

**Conclusions:**

The results suggest that westernized breakfast pattern may confer some protection against metabolic syndrome in Japanese. The causality of these associations needs to be confirmed.

## Introduction

The metabolic syndrome is a cluster of risk factors including obesity, glucose intolerance, insulin resistance, dyslipidemia, and hypertension that increases the risk for cardiovascular disease and type 2 diabetes [1,2]. A striking increase in the number of people with the metabolic syndrome has taken place worldwide [3]. Particularly, among middle aged or older Japanese, one in two men and one in five women were either strongly suspected to have metabolic syndrome or possible metabolic syndrome [4]. Identification of specific foods and nutrients associated with metabolic syndrome is important for the formulation of dietary recommendation [5]. However, nutrients and foods are consumed in combination and their combined effects may be synergic. In this context, dietary pattern analysis, which focuses on a combination of several foods, can provide supplementary data to link diet to disease risk [6,7].

Previous studies that have identified dietary patterns and investigated their association with metabolic syndrome have been reported mainly from US [8,9], North America [10], Samoa [11], Europe [12,13], or Middle East [14-16]. These studies have found that dietary patterns characterized by high intakes of vegetables, fruits, and fish were inversely associated with metabolic syndrome [9,13,15], whereas dietary patterns characterized by high intakes of red meat, processed meat, refined grains and fried foods were associated with increased risk [8,10,12,13,15]. Dietary habits in Asians including Japanese are substantially different from those of Westerners [17]. In contrast to the findings of Western studies, Korean studies [18-21] did not detect a significant association between dietary pattern characterized by meat and alcohol and metabolic syndrome. We are not aware of any study on this issue in Japan, where traditional foods like rice, fish, and soy products are still widely consumed [4], in spite of marked changes in diet over several decades. Interestingly, dietary pattern studies among Japanese [22-24] have shown a protective, rather than positive, association of a diet featured by some western foods including bread and dairy products with prevalence of glucose tolerance abnormalities including type 2 diabetes [22], impaired glucose status measured by A1c [23], and high blood pressure [24]. These observations are not compatible with the general belief that westernization in diet increases risk of cardiometabolic diseases. To further explore this issue, we examined the association of dietary patterns with metabolic syndrome and its components in a Japanese working population.

## Materials and methods

### Study procedure

Data used in this study are derived from a 2006 health survey among employees of two municipal offices in northeastern Kyushu, Japan, as described elsewhere [25,26]. In brief, at the time of the periodic health examination, all full time workers (n = 601) except those on long sick or maternity leave were invited to the survey. Participants were asked to fill out the survey questionnaires beforehand, which were checked by research staff for completeness and, where necessary, clarified by asking the subjects during the examination. We also obtained data that were routinely collected in the health examination, including anthropometric measurements, biochemical data, information about medical history, and smoking. Blood samples were also collected. The study was approved by the Ethics Committee of the National Center for Global Health and Medicine, Japan, and written informed consent was obtained from each participant.

### Study subjects

Of 601 eligible employees, 547 subjects (323 men and 224 women) aged 21–67 years participated in the survey (response rate 91%). After exclusion of 17 subjects with a history of cardiovascular disease or cancer and 5 subjects with missing information on diet, 525 workers (310 men and 215 women) were included for the analysis of dietary pattern [27]. We further excluded 53 subjects with missing information on blood glucose, triglyceride, HDL cholesterol, and blood pressure, 4 subjects with missing information for any of the covariates used in the main analysis, 11 subjects who had not fasted for at least 8 hours. Some subjects fell into more than one exclusion criteria. Finally, 460 subjects (284 men and 176 women) remained for the analysis of the association between dietary patterns and metabolic syndrome.

### Dietary assessment

Dietary habits during the preceding one month period were assessed by using a validated brief self-administered diet history questionnaire (BDHQ) [28], which consists of five sections: 1) intake frequency of 46 food and non-alcoholic beverage items; 2) daily intake of rice and miso soup; 3) frequency of alcohol drinking and the amount of consumption of each five alcoholic beverage per typical drinking occasion; 4) usual cooking method; and 5) general dietary behavior. Dietary intakes for 58 food and beverage items were estimated using an ad hoc computer algorithm for the BDHQ according to the following procedures. For most items (46 items in section 1 and five alcoholic beverages in section 3), dietary intake was calculated based on the reported intake or drinking frequency. For rice and miso soup, dietary intake was calculated based on the reported number of bowls of rice and cups of miso soup per day. Dietary intake of three food items usually added during cooking, namely salt, oil, and sugar, was estimated according to the diet-history method using the qualitative information in sections 4 and 5 together with the food intakes calculated above. Intakes of table salt and salt-containing seasoning at the table as well as soup consumed with noodles were estimated from answers to the corresponding qualitative questions in section 5. Energy intake and nutrient intake from all dietary sources were estimated with reference to the Standard Tables of Food Composition in Japan [29]. According to the validation study of the BDHQ using 16 day semi-weighted dietary records as the gold standard, Spearman`s correlation coefficients for 58 food and beverage items in 92 women aged 31–69 years and 92 men aged 32–76 years were 0.14 to 0.82 (median:0.44) and 0.22 to 0.83 (median:0.48), respectively [30].

### Anthropometric measurements and lifestyles other than diet

Body height was measured to the nearest 0.1 cm with the subjects standing without shoes. Body weight in light clothes was measured to the nearest 0.1 kg. Body mass index (BMI) was calculated as the body weight (kg) divided by the square of the body height (m^2^). Blood pressure was measured in a sitting position. Smoking status, marital status, job title, job position, and non-occupational physical activity were self-reported. Job title was used to create categories for occupational physical activity; sedentary work included managerial and clerical jobs, whereas physically active work included child-care, school lunch cooking, and technical jobs. Non-occupational physical activity was expressed as daily minutes spent for walking or cycling during commute to or from work and weekly hours engaged in each of five different activities (walking, low-, moderate-, and high-intensity activities, and gardening). The sum of time spent for all the non-occupational activities were expressed in hours per week.

### Biochemical measurements

Blood samples were obtained after an overnight fast during the health checkup. Venus blood was drawn in tube and then taken in a cooler box to a laboratory. Biochemical measurements were performed in an external laboratory (Nishinohon Occupational Health Service Center, KitaKyushu). Triglyceride levels were measured by enzymatic method using L-type Wako HG M reagent (Wako Pure Chemical Industries, Ltd, Tokyo), low high-density lipoprotein (HDL) cholesterol was measured by direct method using Choletest N HDL reagent (Daiichi Pure Chemicals Co., Ltd, Tokyo), plasma glucose levels were measured by enzymatic method using Glucose CII Test (Wako Pure Chemical Industries, Ltd, Tokyo), and the measuring device was an OLYMPUS AU640 (Olympus Optical Co., Ltd, Tokyo).

### Definition of metabolic syndrome

Metabolic syndrome and metabolic risk factors were defined according to a modified version of the criteria of the National Cholesterol Education Program’s Adults Treatment Panel III (NCEP-ATP III) [31]. Waist circumference was not measured at the time of survey; we thus used body mass index (BMI) instead of waist circumference. We regarded those with BMI of 25 kg/m^2^ or over as having obesity component of metabolic syndrome, because this BMI cut-off is recommended by WHO for Asians in defining obesity [32] and has been adopted in previous Asian studies on metabolic syndrome [18,33].

Three or more of the following components were defined as having metabolic syndrome: (1) obesity: BMI ≥25 kg/m^2^; (2) high triglycerides: ≥150 mg/dl; (3) HDL cholesterol: <40 mg/dl in men, <50 mg/dl in women; (4) high blood pressure: systolic blood pressure ≥130 mmHg or diastolic blood pressure ≥85 mmHg; and (5) high fasting glucose: ≥100 mg/dl. Participants reporting the current use of antihypertensive or hyperlipidemia medication, or persons who had a history of diabetes were considered to meet the criteria for high blood pressure, high triglyceride, or high fasting glucose, respectively, irrespective of data measured at checkup. In the present study, anti-hyperlipidemia drug included both cholesterol-lowering drug and triglyceride-lowering drug. Subjects with a history of diabetes included not only those under medication but also those who had previously been treated for diabetes but were not under medication.

### Statistical analyses

We performed principal component analysis on the basis of energy-adjusted intakes by using a density method (amount of food intake per 1000 kcal of energy) [34] of 52 food and beverage items excluding six items (sugar added to coffee or black tea, three items usually added during cooking (salt, oil, and sugar), table salt and salt-containing seasoning at the table, and soup consumed with noodles) to derive dietary patterns. The factors were rotated by orthogonal transformation (varimax rotation) to maintain uncorrelated factors and greater interpretability. We considered the eigenvalues, the scree test, and the interpretability of the factors to determine the number of factors to retain. Three dietary patterns were identified. Dietary patterns were named according to the food items showing high loading (absolute value) on three factors. The factor scores for each dietary pattern and for each individual were calculated by summing up the intakes of food items weighted by their factor loadings. Factor scores were categorized into tertiles.

We presented data for men and women combined because we found no effect modification by sex on the association between the three dietary patterns (healthy Japanese, animal food pattern and Westernized breakfast pattern) and metabolic syndrome in the full model (*P* for interaction = 0.76, 0.07, and 0.38, respectively). To show general characteristics of study population, age- and sex-adjusted mean or proportion was presented according to tertile of each dietary pattern score. Trend association between potential confounding variable and dietary pattern score was tested by using the general linear model, with ordinal numbers (1–3) assigned to the tertiles of each dietary pattern.

To evaluate the magnitude of the association between specific dietary patterns and metabolic syndrome and its components (obesity, high triglycerides, low HDL cholesterol, high blood pressure, and high fasting glucose), we estimated adjusted odds ratio (OR) and 95% confidence interval with multivariable logistic regression models. All the models were adjusted for age (year, continuous), sex, workplace (A or B), marital status (married or unmarried), job position (low or middle and high), occupational physical activity (sedentary or active work), current smoking (yes or no), and non-occupational physical activity (0, >0 to <2, or ≥2 hours/week). Trend association was assessed by assigning ordinal numbers 1–3 assigned to tertile categories of each dietary pattern. We also performed the main analysis by including dietary pattern score as a continuous variable. Two-sided *P* values <0.05 were regarded as statistically significant. All analyses were performed using statistical software STATA version 12.0 (Lakeway Drive, College Station, Texas USA).

## Results

We identified three major dietary patterns by principal component analysis (Table 1). The first factor was named a “healthy Japanese dietary pattern” because it represented high intakes of not only vegetables and fruits but also soy products, mushrooms, and green tea. The second factor was characterized by high intakes of fish and shellfish, meat, processed meat, mayonnaise, and egg, and thus it was named an “animal food pattern.” The third factor represented high intakes of bread, confectioneries, milk and yogurt, mayonnaise, and egg and low intakes of rice, alcohol, and fish, and the pattern was named a “westernized breakfast pattern.” The first to third dietary pattern accounted for 9.9, 5.0, and 4.7% respectively, of the variance in food intakes and totally explained 19.5% of the variability.

**Table 1:** **Factor loading matrix for major dietary patterns identified by principal component analysis**^**a**^

	**Healthy Japanese dietary pattern**	**Animal food pattern**	**Westernized breakfast pattern**
Carrots/pumpkin	0.78	–	–
Mushrooms	0.73	–	–
Green leaves vegetables	0.69	–	–
Cabbage/Chinese cabbage	0.68	0.17	–
Japanese radish/turnip	0.68	–	–
Other root vegetables	0.67	–	–
Tofu/atsuage^b^	0.50	–	-0.16
Seaweeds	0.48	0.16	-0.16
Potates	0.46	–	–
Other fruit	0.37	–	0.21
Persimmons/strawberries/kiwifruit	0.31	–	–
Natto^c^	0.30	–	–
Citrus fruit	0.25	–	–
Green tea	0.22	–	–
100% fruit and vegetable juice	-0.19	–	0.17
Buckwheat noodles	-0.25	0.18	–
Cola drink/soft drink	-0.29	–	0.15
Chinese noodles	-0.44	–	–
Squid/octopus/shrimp/shellfish	-0.16	0.47	-0.21
Canned tuna	–	0.44	–
Lean fish	0.15	0.43	–
Pork/beef	–	0.43	–
Dried fish/salted fish	–	0.41	-0.23
Ham/sausage/bacon	–	0.37	–
Chicken	–	0.37	–
Oily fish	–	0.33	-0.29
Lettuces/cabbage (raw)	0.21	0.30	0.17
Tomatoes	0.19	0.29	–
Mayonnaise/dressing	–	0.29	0.28
Small fish with bones	0.20	0.28	-0.23
Liver	–	0.27	–
Egg	–	0.21	0.21
Black tea/oolong tea	–	0.19	–
Other pickles	–	0.18	–
Rice	–	-0.67	-0.34
Bread	–	–	0.57
Western-type confectioneries	–	–	0.56
Milk and yogurt	–	–	0.36
Rice crackers/rice cake/okonomiyaki^d^	–	–	0.36
Japanese confectioneries	–	–	0.36
Ice cream	-0.19	–	0.32
Miso soup	–	–	-0.18
Pickled green leaves vegetables	0.18	0.18	-0.24
Sake	–	–	-0.26
Beer	-0.21	–	-0.41
Shochu	-0.24	–	-0.45

^a^Factor loading less than ±0.15 is represented by a dash for simplicity.

Omitted in the table were food items with factor loadings less than 0.15 for all dietary patterns (coffee, Japanese wheat noodles, low-fat milk and yogurt, spaghetti and macaroni, whisky and wine).

^b^Deep-fried tofu.

^c^Fermented soybeans.

^d^Meat/fish and vegetables pancake.

Table 2 shows the characteristics of the study subjects according to the tertiles of dietary pattern scores. Subjects with a higher score for the healthy Japanese dietary pattern were more likely to be old, women, and physically active, but were less likely to be smokers. Subjects with a higher score for the animal food pattern were more likely to be engaged in sedentary work and under medication for hypertension and have history of diabetes mellitus. Both healthy Japanese and animal food patterns were positively associated with salt and calcium intake but inversely associated with rice intake. Regarding the westernized breakfast pattern, participants with a higher score tended to be younger and women, engaged in a low job position, but were less likely to be smokers and engaged in sedentary work. Westernized breakfast pattern were positively associated with calcium intake, but inversely associated with alcohol, salt, and rice intake.

**Table 2:** **Characteristics of the study participants adjusted for age and sex by tertile of each dietary pattern score**^**a**^

	**Healthy Japanese dietary pattern**				**Animal food pattern**				**Westernized breakfast pattern**			
	**T1 (low)**	**T2**	**T3 (high)**	**Trend *P***^**a**^	**T1 (low)**	**T2**	**T3 (high)**	**Trend *P***^**a**^	**T1 (low)**	**T2**	**T3 (high)**	**Trend *P***^**b**^
Number of subjects	154	153	153		154	153	153		154	153	153	
Age (mean ± SE, year)	43.0 ± 0.8	44.2 ± 0.8	46.6 ± 0.9	<0.01	45.4 ± 0.7	44.6 ± 0.5	43.7 ± 0.7	0.13	47.1 ± 0.7	44.6 ± 0.4	42.0 ± 0.7	<0.01
BMI (mean ± SE, kg/m^2^)	22.8 ± 0.3	22.6 ± 0.3	22.9 ± 0.3	0.86	22.6 ±0.3	22.6 ±0.3	23.1 ± 0.3	0.23	27.7 ± 0.3	22.8 ± 0.3	22.8 ± 0.3	0.87
Workplace (A, %)	41.2	29.2	20.9	<0.01	18.7	30.9	41.4	<0.01	23.9	27.7	39.5	<0.01
Women (%)	9.5	34.7	73.5	<0.01	41.2	35.5	40.9	0.84	21.4	41.0	55.4	<0.01
Married (%)	72.6	76.5	78.9	0.18	73.5	80.8	73.5	0.97	77.5	74.3	76.1	0.86
Sedentary work (%)	80.9	87.5	80.3	0.74	76.3	85.8	86.7	0.01	88.0	81.1	79.6	0.04
Job position (low, %)	54.5	55.1	58.1	0.75	50.6	56.3	61.3	0.44	52.2	50.7	64.9	0.03
Non-occupational physical activity^c ^(≥2 h/week, %)	23.8	29.1	38.9	0.03	30.3	25.2	36.0	0.11	28.7	36.7	25.9	0.49
Under medication for hypertension (%)	1.0	3.8	1.6	0.59	1.6	0.7	4.4	0.04	2.9	1.6	1.7	0.35
History of diabetes mellitus (%)	0.5	1.1	1.9	0.13	0.5	0.6	2.3	0.04	0.8	1.2	0.9	0.79
Current smoker (%)	24.7	15.4	13.9	0.01	17.7	18.6	16.2	0.79	27.0	19.6	7.4	<0.01
Dietary intake (mean ± SE)												
Alcohol (g/day)	237 ± 4	151 ± 5	94 ± 4	0.07	168 ± 7	158 ± 6	157 ± 6	0.72	311 ± 7	134 ± 4	26 ± 2	<0.01
Energy (kcal/day)	1630 ± 43	1780 ± 39	1868 ± 43	<0.01	1765 ± 40	1757 ± 40	1755 ± 40	0.86	1701 ± 41	1825 ± 40	1752 ± 40	0.41
Salt (g/1000 kcal/day)	5.5 ± 0.1	5.6 ± 0.1	5.9 ± 0.1	<0.01	5.0 ± 0.1	5.6 ± 0.1	6.4 ± 0.1	<0.01	5.8 ± 0.1	5.6 ± 0.1	5.5 ± 0.1	0.03
Calcium intake (mg/1000 kcal/day)	222 ± 7	260 ± 6	305 ± 7	<0.01	225 ± 6	265 ± 6	296 ± 6	<0.01	238 ± 7	271 ± 7	277 ± 7	<0.01
Rice (g/1000 kcal/day)	188 ± 6	185 ± 6	167 ± 6	0.03	231 ± 4	178 ± 4	131 ± 4	<0.01	203 ± 5	193 ± 5	144 ± 5	<0.01

Abbreviation: BMI, body mass index; SE, standard error.

^a^All data are given as age- and sex-adjusted mean values or percentages. Mean age was sex-adjusted; percentage of women was age-adjusted.

^b^On the basis of general linear model, assigning ordinal numbers 1–3 to tertile categories of each dietary pattern.

^c^Physical activity was expressed as the sum of weekly hours spent for sport activity, as well as walking and cycling on commuting to and from work.

Table 3 shows odds ratio of metabolic syndrome and components of metabolic syndrome according to categories of each dietary pattern score. The prevalence of metabolic syndrome among men, women, and both sexes combined was 18%, 4%, and 13%, respectively. Westernized breakfast pattern was inversely associated with prevalence of metabolic syndrome (*P* for trend = 0.02) after controlling for age, sex, workplace, occupational physical activity, job position, marital status, and non-occupational physical activity. Subjects in the highest tertile had a 61% lower odds (OR, 0.39; 95% CI: 0.16-0.95) of having metabolic syndrome than those in the lowest tertile. Westernized breakfast pattern was also inversely associated with high blood pressure (*P* for trend = 0.049). Animal food pattern was not significantly associated with the odds of having metabolic syndrome; however, it was positively associated with high blood glucose (*P* for trend = 0.03) and high blood pressure (*P* for trend = 0.05). The healthy Japanese dietary pattern was neither associated with metabolic syndrome nor its components.

**Table 3:** **Multivariable-adjusted OR (95% CI) for metabolic syndrome and its components across tertile of major dietary pattern scores **^**a**^

	**Number of cases, n (%)**	**Healthy Japanese dietary pattern**			**Trend *P***^**b**^	**Animal food pattern**			**Trend *P***^**b**^	**Westernized breakfast pattern**			**Trend *P***^**b**^
		**T**_**1 **_**(low)**	**T **_**2**_	**T**_**3 **_**(high)**		**T**_**1 **_**(low)**	**T **_**2**_	**T**_**3 **_**(high)**		**T**_**1 **_**(low)**	**T **_**2**_	**T**_**3 **_**(high)**	
Number of Subjects	460	154	153	153		154	153	153		154	153	153	
Metabolic syndrome^c^	59 (13)	1.00	1.43	1.35	0.43	1.00	1.03	1.54	0.25	1.00	0.50	0.39	0.02
			(0.71 - 2.88)	(0.55 - 3.30)			(0.48 - 2.18)	(0.73 - 3.24)			(0.24 - 1.02)	(0.16 - 0.95)	
Obesity component	114 (25)	1.00	1.05	1.20	0.59	1.00	0.82	0.94	0.85	1.00	0.97	0.89	0.72
			(0.61 - 1.81)	(0.63 - 2.31)			(0.47 - 1.42)	(0.54 - 1.63)			(0.56 - 1.65)	(0.49 - 1.63)	
High fasting blood glucose	90 (20)	1.00	0.66	0.67	0.26	1.00	1.48	2.08	0.03	1.00	0.65	0.58	0.09
			(0.35 - 1.24)	(0.31 - 1.45)			(0.77 - 2.84)	(1.09 - 3.98)			(0.35 - 1.18)	(0.30 - 1.21)	
High triglyceride	80 (17)	1.00	1.27	0.37	0.16	1.00	0.98	1.45	0.28	1.00	0.86	0.65	0.27
			(0.69 - 2.32)	(0.14 - 0.98)			(0.50 - 1.93)	(0.74 - 2.83)			(0.46 - 1.61)	(0.30 - 1.39)	
Low HDL cholesterol	40 (9)	1.00	1.52	1.20	0.65	1.00	0.53	0.93	0.92	1.00	2.21	1.77	0.25
			(0.67 - 3.45)	(0.43 - 3.37)			(0.22 - 1.29)	(0.42 - 2.07)			(0.93 - 5.25)	(0.67 - 4.69)	
High blood pressure	128 (28)	1.00	1.02	1.37	0.39	1.00	1.07	1.78	0.050	1.00	0.65	0.54	0.049
			(0.57 - 1.80)	(0.69 - 2.74)			(0.60 - 1.92)	(1.01 - 3.17)			(0.35 - 1.12)	(0.31 - 0.99)	

^a ^Adjusted for age (year, continuous), sex, work place (A or B), occupational physical activity (sedentary or active work), job position (low or medium and high), marital status (married or unmarried), non-occupational physical activity (0, >0 to <2 hours/week or ≥2 hours/week), current smoking (yes or no).

^b ^Based on multiple logistic regression analysis, with ordinal numbers 1–3 assigned to tertile categories of each dietary pattern.

^c ^Metabolic syndrome is defined as presence of at least 3 of the following criteria.

Obesity component (BMI ≥25 kg/m^2^), high fasting blood glucose (≥100 mg/dl), high triglycerides (≥150 mg/dl), low-high-density lipoprotein (HDL) cholesterol (<40 mg/dl in men and <50 mg/dl in women), high blood pressure; systolic blood pressure (SBP ≥ 130 mm Hg) or diastolic blood pressure (DBP ≥85 mm Hg).

An exclusion of subjects with history of diabetes (n = 12) and current medication of hypertension (n = 17) and hyperlipidemia (n = 1) did not materially change the results: a significant inverse association between westernized breakfast pattern and metabolic syndrome (*P* for trend = 0.012), while no association with healthy Japanese pattern and animal food pattern (*P* for trend = 0.21 and 0.46, respectively) (data not shown in Table).

In multivariate logistic regression analyses including each dietary pattern score as a continuous variable, westernized breakfast pattern was significantly and inversely associated with metabolic syndrome (β = -0.40, *P* = 0.023), but the animal food and healthy Japanese dietary patterns were not appreciably associated with metabolic syndrome (β = 0.10, *P* = 0.54 and β = 0.22, *P* = 0.25, respectively).

## Discussion

In this cross-sectional study of Japanese working population, three major dietary patterns were identified. Among these dietary patterns, the westernized breakfast pattern was associated with decreased prevalence of metabolic syndrome and high blood pressure. The animal food pattern, which was not associated with metabolic syndrome, was positively associated with high fasting blood glucose. The healthy Japanese dietary pattern was not appreciably associated with metabolic syndrome or its components. To our knowledge, this is the first study among Japanese populations to address the association between dietary patterns and metabolic syndrome.

Dietary patterns similar to westernized breakfast pattern in the present study have been reported in previous studies in Japan [23,35]. Because this pattern is characterized by low intakes of traditional Japanese foods like rice, miso soup, soy product, and fish, but high intakes of bread and dairy foods, it seems to represent westernization of diet, especially breakfast. The protective association of westernized breakfast pattern with metabolic syndrome agrees with findings in previous Japanese studies, where similar dietary patterns are associated with lower prevalence of glucose tolerance abnormalities including type 2 diabetes in middle-aged men [22] and impaired glucose status measured by A1c in general population [23]. The present finding is also consistent with our observation of an inverse association between westernized breakfast pattern and serum C-peptide concentrations [36], a marker of insulin resistance. The protective association with westernized breakfast pattern may represent beneficial effects of some foods or nutrients contributing to the dietary pattern. In a previous Japanese study, milk and dairy food consumption, an important contributor to the westernized breakfast pattern in the present study, was inversely associated with the numbers of metabolic syndrome components [37]. Moreover, in cross-sectional [38] and prospective [8,39] studies among non-Japanese populations, dairy product consumption has been protectively associated with metabolic syndrome. The westernized breakfast pattern in the present study was characterized by lower intakes of alcoholic beverages and subjects with higher westernized breakfast pattern score consumed much lower amount of alcohol (Table 2). According to a meta-analysis [40], favorable metabolic effect of alcohol drinking was confined to women consuming less than 20 g/d of alcohol and to men consuming less than 40 g/d. Higher intake of confectionaries, another feature of westernized breakfast pattern, could deteriorate metabolic profiles, but its effects may not be so strong as to negate the protective role of this dietary pattern against metabolic syndrome.

The present finding of an inverse association of westernized breakfast pattern and high blood pressure agrees with a finding in a Japanese study [24], in which higher score of western dietary pattern, similar to the westernized breakfast pattern, was associated with lower blood pressure. In our study population, persons in the highest tertile of the westernized breakfast pattern consumed less amount of salt than those in the lowest tertile. Because high salt intake is a known risk factor for hypertension [41], the protective association of westernized breakfast pattern against high blood pressure may be ascribed, at least in part, to the lower salt intake in persons with a higher score of westernized breakfast pattern. Additionally as cited earlier, foods or nutrients contributed to westernized breakfast pattern such as milk and yogurt, which are rich source of calcium, have also been shown to decrease blood pressure [42].

In the present study, the animal food pattern, characterized by high intakes of fish and shellfish, meat, and processed meat, was not significantly associated with prevalence of metabolic syndrome. This finding is in agreement with the majority of East Asian studies, in which animal food pattern (named as western pattern or high protein/fat pattern or alcohol pattern) was not associated with prevalence of metabolic syndrome [18,19,21]. At variance with our study, dietary patterns featured by greater intake of animal foods have been consistently associated with higher prevalence of metabolic syndrome in Western populations [8,10,12,13]. Compared with the Western populations, Japanese consume much greater amount of fish but less amount of meat [17]. Therefore, the discrepancy in results for animal food dietary pattern may be ascribed to the difference in types of animal foods commonly consumed as well as their absolute intake level. The animal food pattern was marginally and positively associated with high blood pressure, a finding that might be ascribed to a greater salt intake associated with this dietary pattern (Table 2). We have no plausible reason for the significant positive association between the animal food pattern and high fasting glucose in our study, but this finding appears to be consistent with a previous Japanese study where the seafood dietary pattern was related to higher prevalence of hemoglobin A1C in men [23]. Animal and human studies have shown that a diet high in salt not only increases blood pressure but also deteriorates insulin metabolism [43,44].

The healthy Japanese dietary pattern, characterized by high intakes of vegetables, fruit, mushrooms, and soy products, was not associated with lower prevalence of metabolic syndrome or its components in the present study population. This finding aligns with those in the US [8], Mexican [10], and Iranian [14] studies reporting no association between healthy dietary pattern and metabolic syndrome. In a Japanese study [37], intakes of vegetable and fruit were not associated with the number of metabolic syndrome components. In contrast, studies in Iran [15] and Korea [18] reported a lower prevalence of metabolic syndrome in persons with a higher score of healthy dietary pattern. It should be noted that although healthy Japanese dietary pattern has been characterized by high intakes of fish and shellfish in previous Japanese studies [22-24,45,46], only lean fish and small fish with bones were modestly associated with the healthy Japanese dietary pattern in our study (factor loading: lean fish 0.15; small fish with bones, 0.20). This may partly explain the lack of a protective association with healthy Japanese pattern in the present study population.

Major strengths of the present study include the high study participation rate (91%), and the adjustment for strong potential cofounding variables. Despite these strengths, our study had some limitations that warrant mention. First, an association derived from a cross-sectional study does not necessarily indicate causality. Second, due to the relatively small sample size, statistical power might not be sufficient to detect a moderate association. Third, we used BMI instead of waist circumference to define metabolic syndrome. However, we confirmed that BMI is highly correlated with waist circumference (r = 0.89) in a follow up study, in which both BMI and waist circumference were measured. Fourth, the awareness of previously diagnosed hypertension, hyperlipidemia, and diabetes might have altered dietary patterns, leading to a failure to detect actual associations. Nevertheless, participants under medication for hypertension (4.1%) and hyperlipidemia (0.6%) and with a history of diabetes (2.6%) were few in our study population and we obtained similar results after excluding these subjects. Fifth, although we used a validated dietary questionnaire (BDHQ), some food items showed a low validity; Spearman`s correlation coefficients for green and yellow vegetables, potatoes, seaweeds, and fish and shellfish were 0.37, 0.17, 0.17, and 0.41, respectively, in women and the corresponding correlation coefficients in men were 0.28, 0.21, 0.32, and 0.29. This may be a reason for the lack of an apparent association for the healthy Japanese and animal food patterns. Sixth, principal component analysis has limitation that stems from several subjective decisions in deciding the nature of the components that have been extracted [7,47]. Finally, our study was conducted among municipal employees and hence the results obtained may not be applied to the general population.

In conclusion, results of the present study among Japanese employees suggest that a dietary pattern characterized by high consumption of bread, confectionaries, and milk and yogurt but low consumption of rice and alcohol may be associated with low prevalence of metabolic syndrome. The observed association requires confirmation in prospective studies.

## Abbreviations

BDHQ: Brief self-administered diet history questionnaire; BMI: Body mass index; HDL cholesterol: High-density lipoprotein cholesterol.

## Competing interests

The authors declare that they have no competing interests.

## Authors’ contributions

The authors responsibilities are as follows- SA and AN: conducted data analysis; SA: drafted the manuscript and had primary responsibility for final content; TM: provided statistical expertise, extensively reviewed, and edited the manuscript; and all authors: were involved in interpretation of results and revision of the manuscript and approved the final version of the manuscript.
